# Prevalence and Genetic Characterization of *Blastocystis* in Sheep and Pigs in Shanxi Province, North China: From a Public Health Perspective

**DOI:** 10.3390/ani13182843

**Published:** 2023-09-07

**Authors:** Chang-Ning Wei, Rui-Lin Qin, Zhen-Huan Zhang, Wen-Bin Zheng, Qing Liu, Wen-Wei Gao, Xing-Quan Zhu, Shi-Chen Xie

**Affiliations:** 1 Laboratory of Parasitic Diseases, College of Veterinary Medicine, Shanxi Agricultural University, Jinzhong 030801, China; changningwei0113@163.com (C.-N.W.); q540604070@163.com (R.-L.Q.); zzh13834778507@163.com (Z.-H.Z.); wenbinzheng1@126.com (W.-B.Z.); lqsxau@163.com (Q.L.); sxndgaowenwei@163.com (W.-W.G.); 2 Key Laboratory of Veterinary Public Health of Higher Education of Yunnan Province, College of Veterinary Medicine, Yunnan Agricultural University, Kunming 650201, China; 3 Research Center for Parasites & Vectors, College of Veterinary Medicine, Hunan Agricultural University, Changsha 410128, China

**Keywords:** *Blastocystis*, subtype, prevalence, sheep, pig, Shanxi province

## Abstract

**Simple Summary:**

*Blastocystis* is one of the most prevalent parasites, which can infect humans and many animal species worldwide, and the infection can result in public health problems and economic losses. Sheep and pigs are economically important animals in Shanxi province, north China; however, it is yet to be determined whether they are infected with *Blastocystis*. Thus, the present investigation was conducted to reveal the prevalence of *Blastocystis* in sheep and pigs in three representative counties in Shanxi province by examining 492 sheep feces and 362 pig feces using a molecular approach. The overall prevalence of *Blastocystis* in sheep and pigs were 16.26% and 14.09%, respectively. Five subtypes were found in sheep and pigs via DNA sequence analysis, of which ST5 was the dominant subtype in the three study counties. This study is the first to report the prevalence and subtypes of *Blastocystis* in sheep and pigs in Shanxi province. The findings not only extend the geographical distribution of *Blastocystis* but also provide baseline data for the prevention and control of *Blastocystis* infection in humans and animals in Shanxi province.

**Abstract:**

*Blastocystis* is a common zoonotic intestinal protozoan and causes a series of gastrointestinal symptoms in humans and animals via the fecal–oral route, causing economic losses and posing public health problems. At present, the prevalence and genetic structure of *Blastocystis* in sheep and pigs in Shanxi province remains unknown. Thus, the present study collected 492 sheep fecal samples and 362 pig fecal samples from three representative counties in northern, central and southern Shanxi province for the detection of *Blastocystis* based on its SSU rRNA gene. The results showed that the overall prevalence of *Blastocystis* in the examined sheep and pigs were 16.26% and 14.09%, respectively. Sequences analyses showed that four known subtypes (ST5, ST10, ST14 and ST30) in sheep and two subtypes (ST1 and ST5) in pigs were detected in this study, with ST5 being the predominate subtype among the study areas. Phylogenetic analysis showed that the same subtypes were clustered into the same branch. This study reveals that sheep and pigs in Shanxi province are hosts for multiple *Blastocystis* subtypes, including the zoonotic subtypes (ST1 and ST5), posing a risk to public health. Baseline epidemiological data are provided that help in improving our understanding of the role of zoonotic subtypes in *Blastocystis* transmission.

## 1. Introduction

*Blastocystis* is a common intestinal eukaryotic parasite that is frequently detected in feces in a variety of hosts, including mammals, reptiles and birds [1,2,3,4]. Up to now, one to two billion people worldwide have been infected with *Blastocystis* [5]. The prevalence of *Blastocystis* in most developing nations is higher than that in industrialized countries, which is highly related to socio-economic levels, sanitation infrastructures and geographical areas [6]. The infected hosts show different symptoms according to the host’s susceptibility [7]. After infection, asymptomatic or very mild symptomatic infections occur in most individuals, whereas immunocompromised individuals commonly exhibit gastrointestinal symptoms, such as abdominal pain, vomiting, flatulence and urticaria [8,9]. In addition, some reports have shown that *Blastocystis* was a possible risk factor for anemia in pregnant women [10], and the key nutrients needed for pregnancy, such as iron, glucose, lipids, proteins and so on, produced competitive effects. This competition may have a devastating effect on the growth and development of the fetus, resulting in bleeding during pregnancy [11]. At present, the pathogenicity of *Blastocystis* is controversial, and increasing evidence demonstrates that this depends on the interaction with intestinal microbiota, infection subtypes and the host immune response [7]. Previous reports indicated that infectious cysts of *Blastocystis* could persist in the environment (water and soil) for a long period of time when they were shed with the feces, until infecting the next individual via the fecal–oral route [12,13,14]. The World Health Organization’s drinking water quality publication mentions that *Blastocystis* is a water-related pathogen [15], indicating that *Blastocystis* has a significant impact on public health.

Different diagnostic methods for *Blastocystis* show different levels of sensitivity. Traditionally, detection of *Blastocystis* depends on light microscopy examination of fecal smears; however, various morphological forms of *Blastocystis* make diagnosis difficult [9,16]. Polymerase chain reaction (PCR) has been applied to detect the presence and subtype of *Blastocystis* based on the small subunit ribosomal RNA (SSU rRNA) gene, because a PCR-based method is more sensitive and specific than microscopic and immunological methods [17,18,19]. Previously, numerous epidemiologic studies on *Blastocystis* collectively reported that 30 valid subtypes (ST1–ST17, ST21, ST23–ST34) had been detected from humans and animals, of which ST1–4 contributed to over 95% of *Blastocystis* infections in humans [20,21,22]. ST5 was a frequently identified subtype in hoofed animals worldwide, including pigs and sheep, and considered as a potential zoonotic subtype because it was occasionally detected from farmers that had close contact with animals [23]. ST10 was commonly detected in livestock around the world and, recently, it has been detected in Senegalese school children and Thai adults [24,25]. In addition, enzootic subtypes (ST12, ST14, ST26, etc.) were commonly reported, and their zoonotic potential serves to be evaluated in the future when more data are available.

To date, *Blastocystis* has been detected in many animals worldwide [19,26]. Sheep and pigs are economically important animals in Shanxi province, north China, and they provide valuable meat and furs to the market. Infection of sheep and pigs with *Blastocystis* may reduce livestock performance and pose a zoonotic risk [27]. Thus, understanding the transmission characteristics of *Blastocystis* is of significance to local animal husbandry development and public health. At present, there are no data on *Blastocystis* infection in sheep and pigs in Shanxi province, except a report on alpacas [28]. This study reports the prevalence and subtypes of *Blastocystis* in sheep and pigs in Shanxi province for the first time, which contributes to understanding the prevalence, subtype distribution and public health implications of *Blastocystis* in China.

## 2. Materials and Methods

### 2.1. Sampling Sites

**Figure 1:**
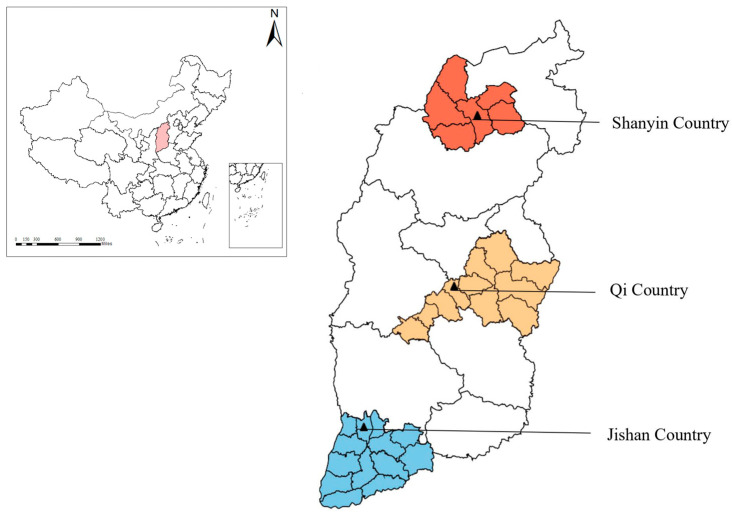
Sampling sites of sheep and pig feces in Shanxi province, north China. The map is based on the standard map service system of the Ministry of Natural Resources of China, drawing review number GS (2019) 1822.

According to the China Statistical Yearbook—2020 (http://www.stats.gov.cn/sj/ndsj/2020/indexeh.htm, accessed on 25 September 2020) and the Shanxi Statistical Yearbook—2019 (http://tjj.shanxi.gov.cn/tjsj/tjnj/nj2019/zk/indexeh.htm, accessed on 25 September 2020), fecal samples were randomly collected from sheep and pigs in three representative counties in Shanxi province, north China. In November 2020, a total of 854 fresh fecal samples (492 sheep feces and 362 pig feces) were collected from central, southern and northern Shanxi province, north China (Figure 1). All samples were directly collected from the rectum with a sterile swab to ensure no cross-contamination and placed in a tube labeled with information (area and age), then kept in a box at a low temperature. Next, all samples were transported to the Laboratory of Parasitic Diseases, College of Veterinary Medicine, Shanxi Agricultural University, and kept in a −20 °C freezer. Before DNA extraction, each sample was thawed at 4 °C.

### 2.2. DNA Extraction and PCR Amplification

The genomic DNA of each fecal sample was extracted using an E.Z.N.A.^®^ Stool DNA Kit (Omega, Bio-Tek Inc., Winooski, VT, USA) according to the manufacturer’s instructions, and stored at −20 °C until the PCR amplification. The *Blastocystis*-positive fecal samples of sheep and pigs were determined by PCR amplification of an ~600 bp fragment of the SSU rRNA gene. The primers BhrDR (5′-GAGCTTTTTAACTGCAACAACG-3′) and RD5 (5′-ATCTGGTTGATCCTGCCAGT-3′) were used in this study, as advised in a previous report [29]. Each 25 μL PCR mixture contained 14.75 μL of ddH_2_O, 2.0 μL of dNTPs, 2.0 μL of MgCl_2_, 2.5 μL of 10 × PCR buffer (Mg^2+^free), 0.5 μL of each primer, 0.25 μL of *Ex*-Taq DNA polymerase (5 U/μL) and 2.5 μL of DNA template. The PCR conditions were as follows: an initial denaturation at 94 °C for 5 min; followed by 30 cycles denaturing at 94 °C for 1 min, annealing at 65 °C for 45 s and extension at 72 °C for 1 min; and a final extension at 72 °C for 10 min. All PCR products were analyzed by electrophoresis in 1.5% agarose gels with ethidium bromide (EB), and the positive products were sent to Sangon Biotech Co., Ltd. (Shanghai, China) for bidirectional sequencing.

### 2.3. Sequencing and Phylogenetic Analysis

The obtained nucleotide sequences were analyzed using the Basic Local Alignment Search Tool (BLAST) on the NCBI website (https://blast.ncbi.nlm.nih.gov/Blast.cgi, accessed on 10 December 2022). The representative sequences of each subtype of *Blastocystis* detected in sheep and pigs in this study were deposited in GenBank under the accession numbers ON062964–ON062987 (sheep) and OM859017–OM859041 (pig), respectively. The phylogenetic analysis was conducted using the neighbor-joining (NJ) method, and Kimura two-parameter (K2P) genetic distances were also calculated using the software MEGA v7.0.26. A bootstrap with 1000 replicates was used to determine the support for the clades generated [28,30]. The outgroup was set to *Proteromonas lacertae* (U37108).

### 2.4. Statistical Analysis

In this study, a chi-squared (χ2) test was used to calculate the statistical difference between the prevalence of *Blastocystis* and risk factors (region and age) using the software SPSS 26.0 (IBM, Chicago, IL, USA). In addition, the odds ratio (OR) and 95% confidence interval (95% CI) were calculated to evaluate the correlation strength between prevalence and test conditions.

## 3. Results

### 3.1. Prevalence of Blastocystis in Sheep and Pigs

In this study, 80 out of 492 sheep fecal samples were detected as positive for *Blastocystis*, with an overall prevalence of 16.26% (80/492) (Table 1). The highest prevalence of *Blastocystis* in sheep was detected in Qi County (32.99%, 32/97), followed by Shanyin County (22.96%, 31/135) and Jishan County (6.54%, 17/260). Statistically significant difference in prevalence were found among the three areas (χ2 = 42.439, *p* < 0.001). However, the prevalence of *Blastocystis* detected in lamb aged less than 6 months (16.11%, 34/211) was only slightly less than that in older sheep aged more than 6 months (16.37%, 46/281); thus, no statistically significant difference was found between the two age groups (χ2 = 0.003, *p* = 0.939).

Meanwhile, 51 out of 362 pig feces samples were successfully amplified and identified as *Blastocystis*-positive in the present study, and the overall *Blastocystis* prevalence in pigs was 14.09% (51/362) (Table 2). In the three study areas, the prevalence of *Blastocystis* in Jishan County was 19.67% (36/183), higher than that in Qi County (17.65%, 12/68) and Shanyin County (2.70%, 3/111). Among age groups, the prevalence of *Blastocystis* in pigs aged less than 6 months (18.57%, 44/237) was significantly higher than that in older pigs aged more than 6 months (5.60%, 7/125). Statistical analysis showed that the prevalence of *Blastocystis* was significantly different among regions (χ2 = 17.31, *p* < 0.001) and ages (χ2 = 11.37, *p* < 0.001).

### 3.2. Subtype Distribution of Blastocystis in Sheep and Pigs

In order to better understand the correlation between prevalence and subtypes of *Blastocystis* in sheep and pigs in study areas, 80 *Blastocystis*-positive sheep samples and 51 *Blastocystis*-positive pig samples were further sequenced and analyzed. Among the 80 sheep-derived samples, four known subtypes (ST5, ST10, ST14 and ST30) of *Blastocystis* were identified (Table 3), with the most prevalent subtype being ST5 (*n* = 40). Notably, ST5 and ST10 subtypes were found in all of the three sampled areas, whereas ST14 was detected in Shanyin County and Jishan County, and ST30 was only detected in Shanyin County. Among the 51 pig-derived samples, two known subtypes (ST1 and ST5) were identified in all of the three study regions (Table 3), and ST5 (*n* = 47) was the predominant subtype.

### 3.3. Phylogenetic Analysis

**Figure 2:**
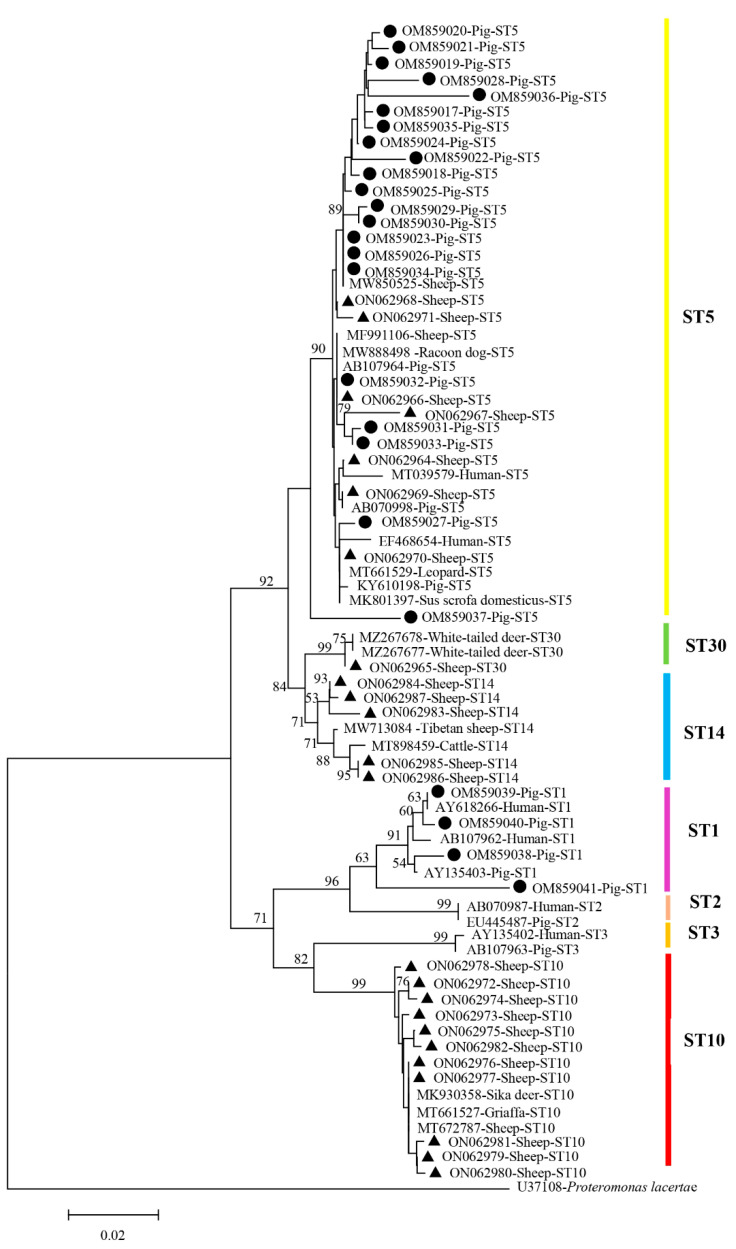
Phylogenetic relationships of *Blastocystis* subtypes based on SSU rRNA sequences. Obtained *Blastocystis* sequences from sheep and pigs in the present study are marked with black triangle (▲) and black circle (●), respectively. The bootstrap value < 50% is hidden.

The *Blastocystis* sequences obtained from sheep and pigs in this study corresponded to the sequences ST1, ST5, ST10, ST14 and ST30 obtained from the GenBank database, while the outgroup was in a single branch (Figure 2). We found that the corresponding ST5 sequence from pigs in the present study was highly similar to that of sheep isolates and human isolates. For example, 100% sequence homology was observed for *Blastocystis* ST5 between a pig isolate (OM859032) and sheep isolate (ON062966). In addition, the obtained sequence of the ST1 subtype (OM859039) from pigs was highly similar to that isolated from humans (AY618266).

## 4. Discussion

*Blastocystis* is an important microorganism, which has been frequently detected in fecal samples from humans and animals worldwide, and the shared *Blastocystis* STs between humans and animals indicate a potential risk of zoonotic transmission to humans [2]. In the present study, the *Blastocystis* prevalence in sheep and pigs in Shanxi province were 16.26% (80/492) and 14.09% (51/362), respectively. The *Blastocystis* prevalence in sheep in Shanxi province was lower than that in Turkey (38.2%, 84/220) [31], the United Arab Emirates (63.6%, 7/11) [32], Italy (81.8%, 9/11) [33], Brazil (33.3%, 1/3) [34], Iran (20.9%, 14/67) [35], and some provinces of China, such as Jiangsu province (24.00%, 18/75) and Shandong province (16.67%, 10/60) [36]. However, the 16.26% *Blastocystis* prevalence in sheep in Shanxi province was higher than that in Anhui province (6.4%, 22/697) and the Qinghai-Tibetan Plateau (8.55%, 53/620) [36,37]. For pigs, *Blastocystis* was highly prevalent worldwide, with a range of prevalence: 12.00% in Slovakia [38], 45.20% in Cambodia [39], 57.89% in Brazil [40], 76.40% in Thailand [41], 76.70% in Australia [39] and 22.89% to 74.80% in many provinces of China [42,43,44]. The differences in the prevalence of *Blastocystis* between different studies may be due to sample size, animal immune status and geographical environment [22,45].

In this study, there were significant differences in *Blastocystis* prevalence in both sheep and pigs among the sampled regions (*p* < 0.001). With regards to three regions, the highest *Blastocystis* prevalence in sheep was detected in Qi County (32.99%, 32/97), followed by Shanyin County (22.96%, 31/135) and Jishan County (6.54%, 17/260). The possible factor for high *Blastocystis* prevalence in Qi County may be its unique geographic location, because it is close to Taiyuan city, the capital of Shanxi province, which has a dense population and the largest transportation network, and high accessibility can easily lead to the spread of pathogens. In addition, different climates may also contribute to the different prevalence of *Blastocystis* in the three study areas. The *Blastocystis* prevalence in pigs in Jishan County was 19.67% (36/183), higher than that in Qi County (17.65%, 12/68) and Shanyin County (2.70%, 3/111), and the prevalence of *Blastocystis* in pigs gradually decreased with increasing latitude (Figure 1). We speculated that the mild and warm climate in the lower latitudes of Jishan County may contribute to the different prevalence of *Blastocystis* compared to the other study areas. The difference in *Blastocystis* prevalence between sheep and pigs in the same area may be due to differences in sampling size, managing differences and different husbandry patterns, as well as the different degrees of susceptibility of sheep and pigs to *Blastocystis* infection.

With regards to the age groups, there was no statistically significant difference in *Blastocystis* prevalence in sheep (*p* = 0.939); however, a statistically significant difference was found in pigs (*p* < 0.001). Our results were consistent with those previously reported in sheep and in pigs [36,46]. The age factor is considered an important factor influencing *Blastocystis* transmission among animals, but this point is still controversial [47]. A study reported that the higher *Blastocystis* prevalence in young pigs might be due to their imperfect immune system [46]. However, a high prevalence of *Blastocystis* was also found in older pigs in different studies [44,48]. Furthermore, host age had no significant effect on *Blastocystis* infection in a previous study [36]. Therefore, it is difficult to explain the discrepancies in *Blastocystis* prevalence between different studies.

The phylogenetic tree revealed that all five subtypes were clustered into their corresponding branches of subtypes. Among the four subtypes (ST5, ST10, ST14, ST30) and two subtypes (ST1, ST5) detected in sheep and pigs in this study, respectively, ST5 was the most prevalent subtype in both sheep (50.0%, 40/80) and pigs (92.2%, 47/51). ST5, a dominant subtype generally identified in hoofed animals [44], has also been detected in humans with close animal contact, highlighting its potential for zoonotic transmission to humans [23,49]. Previous reports indicated that ST10 and ST14 were only detected in ruminants [45,50,51]; however, both ST10 and ST14 were recently detected in Senegalese school children and Thai adults [24,25], suggesting that its transmission dynamics warrant further study. At present, the prevalence and subtype characterization of *Blastocystis* ST30 is still limited, which was only identified in sheep and camel in China since it was detected in deer in the USA for the first time [20,52,53]. The four zoonotic STs (ST1, ST5, ST10, ST14) obtained in this study highlight that both sheep and pigs may play a very prominent role in the transmission cycle of *Blastocystis* zoonotic subtypes.

Parasitic diseases increase the burden of infectious diseases all over the world to a great extent [54]. The transmission of parasites between humans and animals is an issue of public health and veterinary significance. Animals play a critical role in the One-Health Strategy with regards to the prevention and control of zoonotic diseases [55]. *Blastocystis*, as a ubiquitous zoonotic parasite with a worldwide distribution [5], will not only cause irritable bowel syndrome (IBS) but also compete with pregnant women for nutrient elements, resulting in bleeding during pregnancy [11]. Although it is still controversial as to the pathogenicity of *Blastocystis*, the clinical symptoms caused by *Blastocystis* infection still need to be treated. At present, metronidazole (MTZ) is the drug of choice for *Blastocystis* treatment, but it does not have a good power to improve gastrointestinal symptoms [56]. Therefore, prevention remains the top priority to control *Blastocystis* infection. Firstly, other studies have shown that intestinal protozoans can cause zoonotic diseases related to livestock and domestic pets (transmitted from animals to humans) [57], so people in close contact with animals need to take some necessary safety measures, such as hand hygiene and keeping distance with animals [25,39]. Secondly, it is necessary to optimize the breeding environment, especially in terms of fecal cleaning and drinking water safety. Since the fecal–oral route is the principal means of transmission for *Blastocystis* infection, improving the management of the feeding environment can reduce the risk of infection in the external environment. Finally, steps should be taken to increase the molecular epidemiological surveillance of *Blastocystis* in humans and animals with low immune function [58]. The above measures can effectively prevent and control the spread of *Blastocystis*, and then reduce the adverse effects of *Blastocystis* on public health.

## 5. Conclusions

This study reported the prevalence and subtypes of *Blastocystis* in sheep and pigs in Shanxi province for the first time. The prevalence of *Blastocystis* in sheep and pigs were 16.26% and 14.09%, respectively. Four zoonotic subtypes (ST1, ST5, ST10 and ST14) and one species-specificity subtype (ST30) were identified according to *Blastocystis* SSU rRNA sequences, indicating a potential zoonotic transmission risk to humans and other animals. The results of the present study indicate that measures should be taken to reduce the risk of *Blastocystis* infection in humans and animals.

## Figures and Tables

**Table 1:** Factors associated with prevalence of *Blastocystis* in sheep in Shanxi province.

Factor	Category	No. Tested	No. Positive	Prevalence% (95% CI)	OR (95% CI)	*p*-Value
Region	Qi County	97	32	32.99 (23.63–42.35)	7.04 (3.68–13.46)	*p* < 0.001
	Shanyin County	135	31	22.96 (15.87–30.06)	4.26 (2.26–8.04)	
	Jishan County	260	17	6.54 (3.53–9.54)	1	
Age	≤6 M	211	34	16.11 (11.15–21.07)	1	*p* = 0.939
	>6 M	281	46	16.37 (12.04–20.70)	1.02 (0.63–1.65)	
Total		492	80	16.26 (13.00–19.52)		

M: month.

**Table 2:** Factors associated with prevalence of *Blastocystis* in pigs in Shanxi province.

Factor	Category	No. Tested	No. Positive	Prevalence% (95% CI)	OR (95% CI)	*p*-Value
Region	Qi County	68	12	17.65 (8.59–26.71)	7.71 (2.09–28.47)	*p* < 0.001
	Shanyin County	111	3	2.70 (0–5.72)	1	
	Jishan County	183	36	19.67 (13.91–25.43)	8.82 (2.65–29.38)	
Age	≤6 M	237	44	18.57 (13.62–23.52)	3.84 (1.68–8.81)	*p* < 0.001
	>6 M	125	7	5.60 (1.57–9.63)	1	
Total		362	51	14.09 (10.50–17.67)		

M: month.

**Table 3:** Prevalence and subtypes of *Blastocystis* in sheep and pigs in Shanxi province.

Host	Factor	Category	No. Positive/Tested	Prevalence (%)	Subtype (*n*)
Sheep	Region	Qi County	32/97	32.99	ST5 (31), ST10 (1)
		Shanyin County	31/135	22.96	ST5 (8), ST10 (11), ST14 (11), ST30 (1)
		Jishan County	17/260	6.54	ST5 (1), ST10 (13), ST14 (3)
		Subtotal	80/492	16.26	ST5 (40), ST10 (25), ST14 (14), ST30 (1)
Pig	Region	Qi	12/68	17.65	ST5 (11), ST1 (1)
		Shanyin	3/111	2.70	ST5 (2), ST1 (1)
		Jishan	36/183	19.67	ST5 (34), ST1 (2)
		Subtotal	51/362	14.09	ST5(47), ST1(4)
		Total	131/854	15.34	ST5 (87), ST10 (25), ST14 (14), ST1(4), ST30 (1)

ST: subtype.

## Data Availability

The datasets supporting the results of this article have been submitted to GenBank and the accession number shown in the article.
